# LSD1 Overexpression Is Associated with Poor Prognosis in Basal-Like Breast Cancer, and Sensitivity to PARP Inhibition

**DOI:** 10.1371/journal.pone.0118002

**Published:** 2015-02-13

**Authors:** Satoi Nagasawa, Anna S. Sedukhina, Yuko Nakagawa, Ichiro Maeda, Manabu Kubota, Shigeko Ohnuma, Koichiro Tsugawa, Tomohiko Ohta, Marta Roche-Molina, Juan A. Bernal, Ana J. Narváez, Anand D. Jeyasekharan, Ko Sato

**Affiliations:** 1 Department of Translational Oncology, St. Marianna University Graduate School of Medicine, Kawasaki, 216-8511, Japan; 2 Division of Breast and Endocrine Surgery, Department of Surgery, St. Marianna University School of Medicine, Kawasaki, 216-8511, Japan; 3 Department of Obstetrics and Gynecology, St. Marianna University School of Medicine, Kawasaki, 216-8511, Japan; 4 Department of Pathology, St. Marianna University School of Medicine, Kawasaki, 216-8511, Japan; 5 Department of Cardiovascular Development and Repair, Centro Nacional de Investigaciones Cardiovasculares (CNIC), c/Melchor Fernandez Almagro 3, 28029, Madrid, Spain; 6 University of Cambridge, The Medical Research Council Cancer Cell Unit, Hutchison/MRC Research Centre, Hills Road, Cambridge, CB2 OXZ, United Kingdom; 7 Cancer Science Institute of Singapore, National University of Singapore, Centre for Translational Medicine, 14 Medical Drive, #12-01, Singapore, 117599, Singapore; Northwestern University, UNITED STATES

## Abstract

LSD1, a lysine-specific histone demethylase, is overexpressed in several types of cancers and linked to poor outcomes. In breast cancer, the significance of LSD1 overexpression is not clear. We have performed an *in silico* analysis to assess the relationship of LSD1 expression to clinical outcome. We demonstrate that LSD1 overexpression is a poor prognostic factor in breast cancer, especially in basal-like breast cancer, a subtype of breast cancer with aggressive clinical features. This link is also observed in samples of triple negative breast cancer. Interestingly, we note that overexpression of LSD1 correlates with down-regulation of BRCA1 in triple negative breast cancer. This phenomenon is also observed in *in vitro* models of basal-like breast cancer, and is associated with an increased sensitivity to PARP inhibitors. We propose therefore that high expression levels of the demethylase LSD1 is a potential prognostic factor of poor outcome in basal-like breast cancer, and that PARP inhibition may be a therapeutic strategy of interest in this poor prognostic subtype with overexpression of LSD1.

## Introduction

LSD1, lysine specific demethylase 1, removes methyl groups from lysine residues of histone H3, thereby regulating gene expression [1]. For gene repression, LSD1 removes mono- and dimethyl groups from lysine 4 of histone H3 (H3K4) [1]. For gene activation, LSD1 works with androgen/estrogen receptor to remove mono- and dimethyl groups from lysine 9 of histone H3 (H3K9) [2,3]. The control of gene expression by LSD1 has been shown to be vital to multiple processes including organogenesis and stem cell differentiation [4],[5]. In intracellular processes, it has been suggested that LSD1 promotes cell proliferation, survival and epithelial-mesenchymal transition (EMT) [6]. LSD1 is often overexpressed in malignancies and it is linked to poor clinical outcome in cancers of the lung, liver, colon and esophagus [7–10]. Overexpression of LSD1 has been reported in estrogen receptor negative breast cancer, however it is not known whether LSD1 is a prognostic factor of poor outcome in breast cancer [11].

Breast cancer has been classified into four subtypes based on gene expression profile [12]. Basal-like breast cancer, one of the subtypes, does not display hormonal receptors and human epidermal growth factor receptor 2, HER2, suggesting resistance to hormonal therapy and HER2 antagonism [13]. These tumors display an aggressive clinical course, with high relapse rates [13], and are an important area for the development of new therapeutic strategies. Loss of BRCA1, a familial breast cancer susceptible gene, through mutation or epigenetic dysregulation often leads to tumors with a basal-like phenotype [14]. Recent work has implicated LSD1 in this dysregulation of BRCA1 [6]. Wnt signaling is upregulated in basal-like breast cancer, leading to accumulation of the transcriptional repressor Slug (Snail2) [6]. The accumulated transcription repressor targets LSD1 to promoter region of BRCA1 leading to its downregulation [6]. Thus LSD1 may play a critical role in acquiring poor prognostic phenotype in breast, but the relationship between expression of *LSD1* and the clinical outcome has not been demonstrated to date.

Using bioinformatics tools, we predict that LSD1 expression is linked to poor recurrence free survival of patients with breast cancer, especially in the basal-like breast cancer. We have also investigated the relationship between *LSD1* expression and recurrence free survival in 32 samples of triple negative breast cancer and found that *LSD1* is a prognostic factor of poor clinical outcome. Furthermore, we have shown that *LSD1* overexpression is linked to BRCA1 suppression. Therefore, we propose that PARP inhibitors, a novel class of targeted agents with promising activity in *BRCA* mutant tumors, may be effective therapy for basal-like breast cancers with amplified *LSD1* [15].

## Materials and Methods

### 1.1. Bioinformatic analysis

Gene count data from Breast invasive carcinoma TCGA samples (RNA sequencing) were downloaded from TCGA data portal. PAM50 definitions of intrinsic subtype were used to classify breast cancer into subtypes including basal-like, luminal A, luminal B and HER2 enriched cohorts. The quantitation of mRNA expression was performed using All Complete Tumors of Breast invasive carcinoma (TCGA, Nature 2012) dataset [16]. For the analysis of gene expression, raw counts were normalized by Trimmed Mean of M-values (TMM) using the R package “edgeR” and “calcNormFactors” command. For survival analysis, a set of CEL files (GSE1456) were downloaded from GEO database and normalized by MAS5.0 global mean method. Probe set-based signal intensities were natural log transformed and scaled by adjusting the mean intensity to a target signal value of log500. For survival analysis using KMplot, the data was obtained from kmplot (www.kmplot.com) [17].

### 1.2. Statistical Analysis

Differential mRNA expression between two or more groups was analyzed by edgeR. For survival analysis, Gehan-Breslow-Wilcoxon tests were performed, as well as cox proportional hazard models. To compare gene expression of BRCA1 in cancer with high or low LSD1, the Mann Whitney U test was performed. Difference were considered to be significant when the two-tailed p-value was < 0.05. Gehan-Breslow-Wilcoxon tests and Mann-Whiney U test were performed using Graphpad prism. Cox proportional hazard models were fitted using the coxph function (located in the survival library in R).

### 1.3. Study approval

This project was approved by the Clinical Ethics Committee of the St. Marianna University-approval number: 2297-i103, with a waiver of consent granted for the use of archival clinical samples from the St. Marianna University Department of Pathology.

### 1.4. Immunohistochemistry (IHC) and measurement of protein expression

Paraffin tissue sections were cut onto coated slides (3μm) and deparaffinized by routine techniques. Endogenous peroxidase activity was blocked with a 3% H_2_O_2_ in PBS for 5min. For LSD1 staining, the sections were incubated with an anti-LSD1 antibody for 60 min. For BRCA1 staining, antigen retrieval was performed with Antigen Retrieval Solution (pH 9.0) (Nichirei bioscience) at 95°C in a steamer for 40min, followed by the sections were incubated with an anti-BRCA1 antibody for 60 min. Labeling was detected with the Histofine Simple Stain, MULTI (Nichirei Bioscience), following the protocol suggested by the manufacturer, and all sections were counter stained with hematoxylin. For measurement of protein expression, the percentage of positive cells was determined by counting about 500 cells within five high-resolution fields. Immunohistochemical staining (IHC score) was evaluated using the semi-quantitative Remmele scoring system [18], which links the IHC staining intensity (SI) with the percentage of positive cells (PP) (Table 1). High-LSD1 is defined as above average IHC score = 5.

**Table 1:** Criteria of IHC scoring.

**Score**	**Criteria**
Staining Intensity (SI)	0: no staining
	1: weak staining
	2: moderate staining
	3: intensive staining
Percentage of Positive Cells (PP)	0: no positive cells
	1: less than 10%
	2: 11–50%
	3: 51–80%
	4: >80%

### 1.5. Cell culture

MDA-MB-231 and MDA-MB-157 were cultured in L-15 medium supplemented with 10% foetal bovine serum and 1% antibiotic-antimycotic agent at 37°C. HCC70 were cultured in RPMI1640 medium supplemented with 10% foetal bovine serum and 1% antibiotic-antimycotic agents at 37°C with 5% CO_2_.

### 1.6. Plasmids

A construct including cDNA of LSD1 was a kind gift from Dr. Atsushi Yokoyama [19]. The cDNA of LSD1 was subcloned into pcDNA3-myc- vector.

### 1.7. Antibodies

The antibodies and dilution used in this study were: Anti-BRCA1 (07–434) antibody (Millipore, 1:2000 for WB, 1:500 for IHC); Anti-Myc (9E10) antibody (Neomarker, 1μg/μl, 1:1000); Anti-LSD1 (2139) antibody (Cell Signaling Technology, 1:1000); and anti-β-actin (AC-15) antibodies (Sigma, 1:1000).

### 1.8. Western blots

MDA-MB-231 and MDA-MB-157 were transfected with Lipofectamin2000 transfection reagent. HCC70 was electroporated with Cell Line Nucleofector Kit V in Nucleofector (Lonza) (2x10^6^ cells, 2ug of DNA, program P-020). Western blots were done as described previously, briefly 48 hours after transfection, cells were lysed with 0.5% NP-40 lysis buffer (50mM Tris-HCl pH7.5, 150mM NaCl, 0.5% NP-40, 50mM NaF, 1mM DTT, 1mM Na_3_VO_4_, complete protease inhibitor cocktail (Roche) and 1mM PMSF) and resolved by SDS-PAGE [20].

### 1.9. Colony formation assay

Cells were transfected as described above. Twenty-four hours after transfection, cells were plated into 6-well plates at a density of 4000 cells per well. Different doses of Olaparib (a PARP inhibitor, AZD2281, KU-0059436) were added, and the plates were incubated at 37°C for a week. Cells were fixed with 75% methanol in 25% acetic acid for 5 min, and the plates were dried. Colonies were stained with Lillie's crystal violet (2 g crystal violet, 0.8 g ammonium oxalate in 100 ml of 80% ethanol) for 5 min and subsequently washed with water, dried, and measured by ImageQuant LAS 4000 (GE healthcare).

## Results

### 2.1. Increased expression of LSD1 transcripts is observed in basal-like breast cancer.

**Fig 1:**
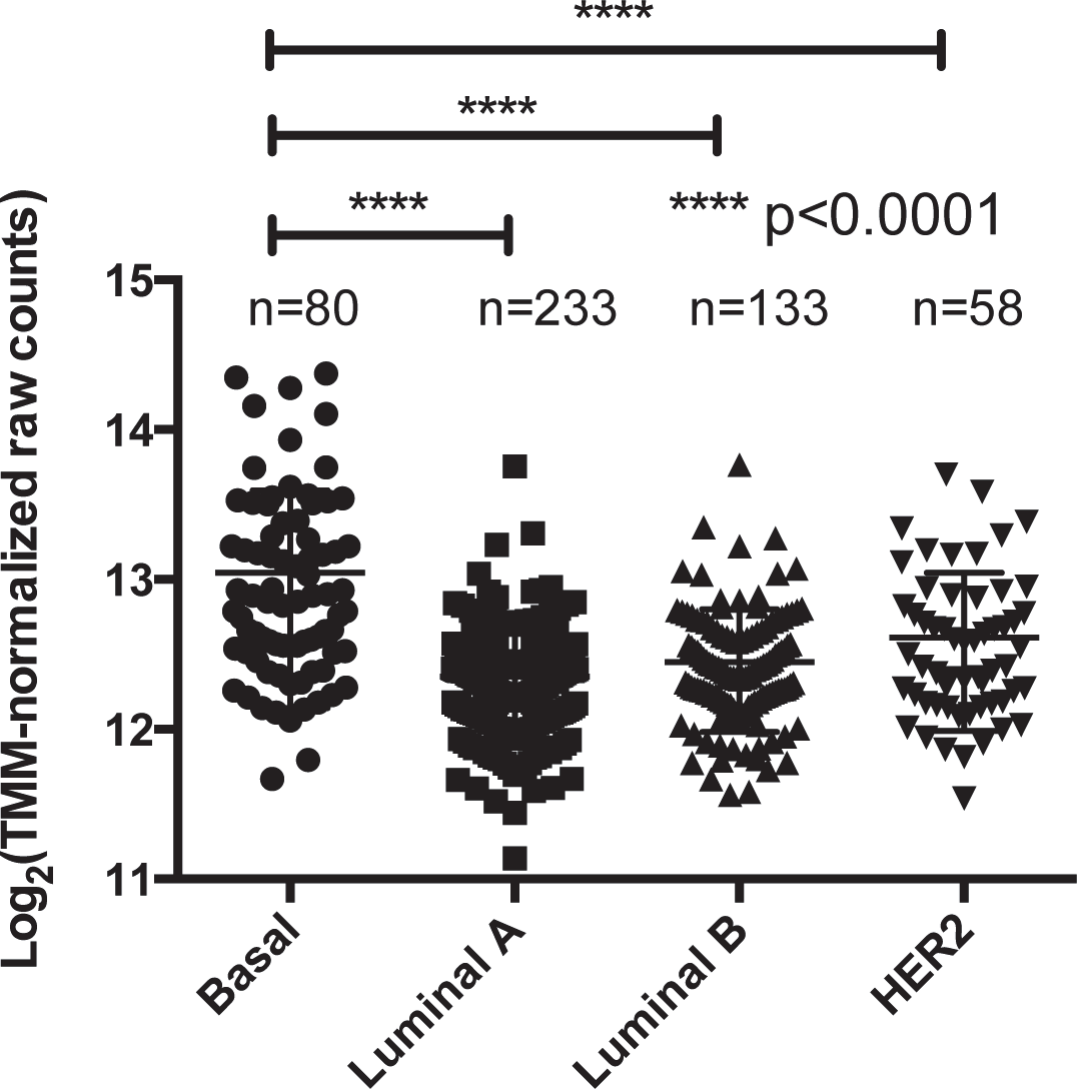
Overexpression of LSD1 is observed only in basal-like breast cancer. This is a plot of LSD1 expression from the TCGA dataset (expression of mRNA as log_2_ TMM-normalized raw counts in the y-axis) plotted across different subtypes of breast cancer (x-axis). Significant differences in mRNA between intrinsic subtypes were calculated using edgeR. The number of samples were as shown in the scatter plots. LSD1 is amplified in basal-like breast cancer when compared to other subtypes, in this dataset.

In order to validate whether *LSD1* is overexpressed in each intrinsic molecular subtypes of breast cancer, we have analyzed *LSD1* mRNA expression data from The Cancer Genome Atlas (TCGA). The data was statistically analyzed by edgeR [21] and the analysis indicates that basal-like breast cancers show a significantly higher amount of LSD1 transcript than the other subtypes. (p<0.0001) (Fig. 1 and Table 2). Although it is known that *LSD1* is overexpressed in ER-negative breast cancer, upregulation of *LSD1* transcripts is not seen in HER2 type breast cancer that is also ER-negative (Fig. 1) [11].

**Table 2:** mRNA expression of LSD1 in subtypes of breast cancer.

**Subtypes**	**p-value**	**q-value**
Basal-like vs Luminal A	7.10E-40	8.97E-39
Basal-like vs Luminal B	3.27E-16	2.40E-15
Basal-like vs HER2	9.12E-06	4.21E-05
Basal-like vs Luminal A vs Luminal B vs HER2	7.39E-38	7.21E-37

False discovery rate calculated by edgeR are also shown as q-value.

### 2.2. mRNA level of *LSD1* is a prognostic factor in basal-like breast cancer

**Fig 2:**
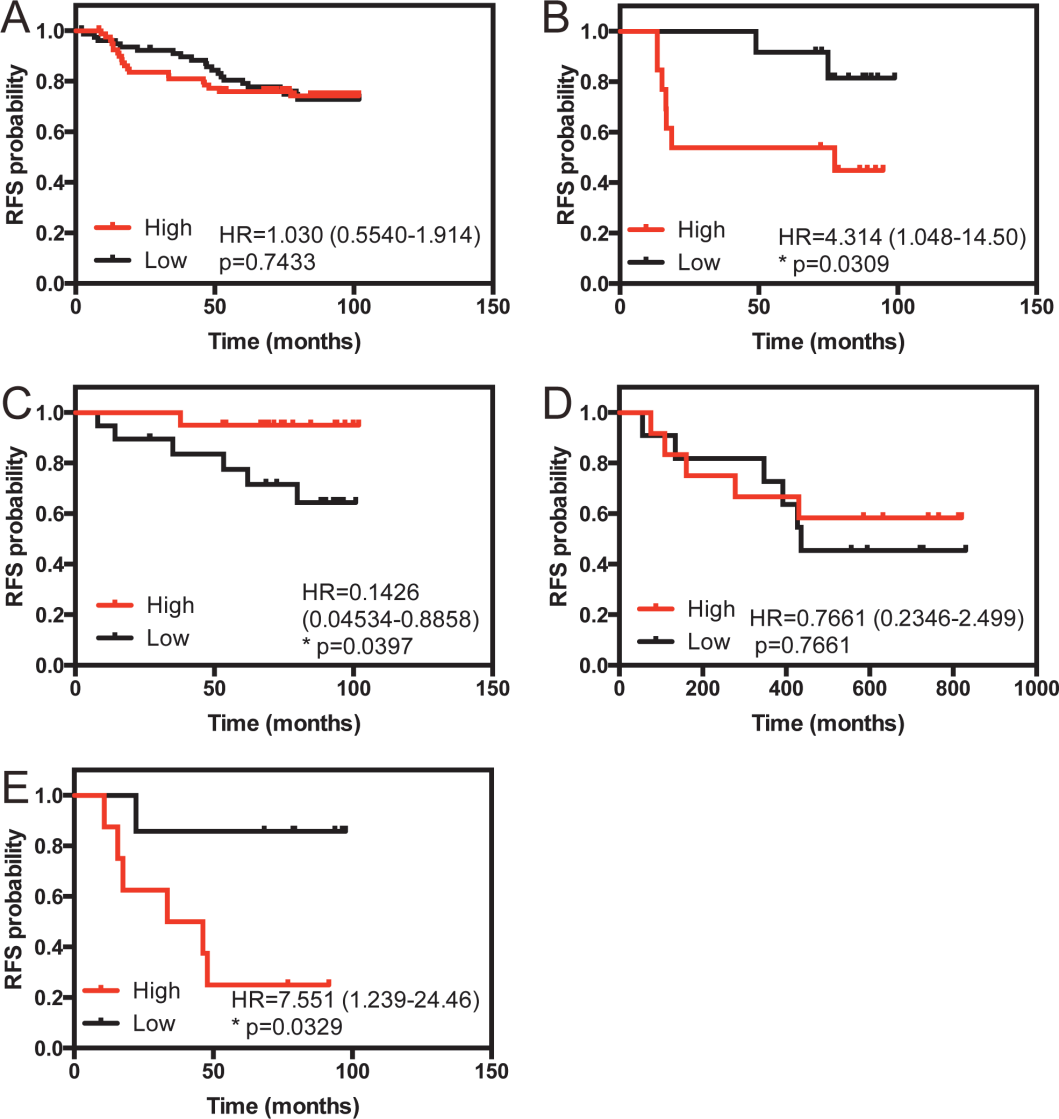
Overexpression of LSD1 is linked to poor outcome in basal-like breast cancer. Effect of LSD1 overexpression (as defined by scores > median) on recurrence free survival (RFS) across all or for each subtype of breast cancer are shown (overall breast cancer, n = 159 (A), basal-like, n = 25 (B), luminal A, n = 39 (C), luminal B, n = 23 (D) and HER2, n = 15 (E)). Overexpression of LSD1 transcripts shows significant shorter RFS in basal-like or HER2 type breast cancer (p = 0.0309, p = 0.0329, respectively). P-value was calculated using Gehan-Breslow-Wilcoxon test.

Among the intrinsic subtypes of breast cancer, basal-like and HER2 type breast cancer display the most aggressive clinical features, but still lack defined molecular prognostic factors [13]. *LSD1* is an established prognostic factor of poor outcome in several types of malignancies [7] [8] [9] [10]. In breast, the impact of *LSD1* on prognosis is not known. As shown in Fig. 1, *LSD1* expression status is altered only in basal-like breast cancer. This prompted us to investigate the correlation between *LSD1* level and the clinical outcome. We have analyzed a single large dataset (n = 159) available in GEO of recurrence free survival (RFS) to evaluate whether *LSD1* overexpression is a prognostic factor in each intrinsic subtype of breast cancer [22]. The analysis indicates that cancer with high *LSD1* transcripts shows a trend to shorter recurrence free survival (RFS) in basal-like breast cancer with a hazard ratio (HR) of 4.314 (Fig. 2B). Interestingly, high level of LSD1 transcripts is related to good prognosis in luminal A type breast cancer (HR = 0.1426) and poor prognosis in HER2 type breast cancer (HR = 7.551) (Fig. 2C). To expand these analyses, we have also looked at recurrence free survival data available at KMplot (www.kmplot.com) that contains large number of samples including the GEO dataset [17]. The analysis indicates that cancer with high *LSD1* transcripts shows a trend to shorter recurrence free survival (RFS) when all subtypes of breast cancer are pooled (P = 0.024) (S1A Fig.). However, in basal-like breast cancer, there is a clearer link to poor outcome with a hazard ratio of 1.39 (S1B Fig.). Thus, these results indicate that increased expression of *LSD1* transcripts could serve as a prognostic factor for poor outcome in basal-like breast cancer.

### 2.3. Protein level of LSD1 is a prognostic factor in triple negative breast cancer

**Fig 3:**
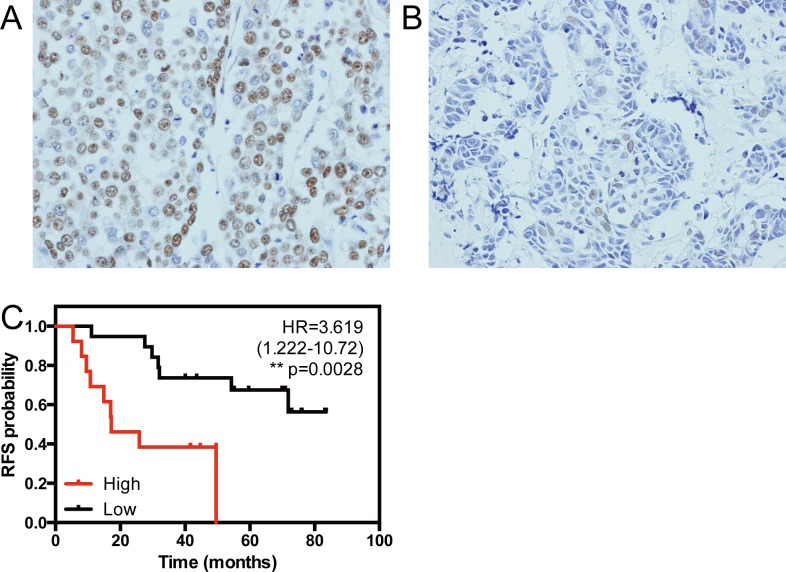
Overexpression of LSD1 is linked to poor outcome in triple negative breast cancer. Representative images of immunohistochemistry of LSD1 in 20 clinical samples are shown (A and B, LSD1 high and low, respectively). The Kaplan Meir curve compares the recurrence free survival of cancer with high or low level LSD1 protein products (C). Definition of LSD1 high is samples that have IHC scores above 5.

Breast cancers have traditionally been classified on the basis of immunohistochemical reactivity to Estrogen Receptor (ER), Progesterone Receptor (PR) and HER2. Tumors which lack reactivity to all these three markers are termed ‘triple negative’ breast cancer, which constitute a clinically aggressive subset [23]. Molecular profiling has shown significant overlap between the gene-expression based subtype ‘basal-like’ and the immunohistochemical ‘triple negative’ subtypes of breast cancer [12] [24] [25]. To extend the results from our bioinformatic analysis, we have checked the impact of expression of *LSD1* protein product on prognosis in triple negative breast cancer (TNBC), given the similarity in clinical features to basal-like breast cancer. For this study, we used samples from 32 patients at St. Marianna University hospital with clinical information, diagnosed and treated between 2007 and 2011. The median follow-up period is 1279 days. The clinical information of the patients is shown in (Table 3). Eleven samples had high level of *LSD1* protein (IHC score > 5, see materials and methods), which correlate with shorter recurrence free survival, showing a hazard ratio of 3.619 (Fig. 3A-C, Table 4). We have also used the cox proportional hazard model to adjust for the effect of age. This analysis also shows that LSD1 is a prognostic factor of poor clinical outcome with a hazard ratio of 3.688 (Table 5). Thus, in TNBC, high expression of the protein product of *LSD1* is a prognostic factor of poor outcome, extending our observations on the link between LSD1 transcript levels and outcome in basal-like breast cancer.

**Table 3:** Clinical information of triple negative breast cancer.

**Average age**	**54.3 (28–83) 54 (median)**	
Chemotherapy	Neo-adjuvant	21
	Adjuvant	11
Clinical Stage	IA	7
	IIA	7
	IIB	11
	IIIA	3
	IIIB	4
Lymph Nodes	Negative	20
	Positive	12
Relapse	Negative	16
	Positive	16

**Table 4:** Protein expression of BRCA1 and LSD1 in triple negative breast cancer.

**BRCA1**	**LSD1**
2	2
0	12
0	12
2	6
6	4
0	3
1	8
2	6
2	2
6	2
2	2
2	3
0	4
0	3
6	3
6	2
0	2
1	2
9	2
2	0
3	8
1	6
0	12
2	1
2	12
8	2
5	4
0	12
4	3
3	12
9	4
6	4

IHC score are shown.

IHC score = SI*PP (see material and methods)

**Table 5:** Cox proportional hazard model for clinical samples.

	**univariate**			**adjusted**		
	HR	95% C.I.	p-value	HR	95% C.I.	p-value
LSD1	3.619	1.222–10.72	0.0203	3.688	1.232–11.040	0.0197
Age	1.002	0.966–1.032	0.912	1.005	0.962–1.029	0.7573

Age was evaluated as a continuous variable.

### 2.4. Overexpression of LSD1 suppresses BRCA1 expression and is associated with sensitivity to PARP inhibition

Wnt signaling is upregulated in basal-like breast cancer [6]. Canonical Wnt signaling promotes degradation of GSK-3β by ubiquitin-proteasome system, which in turn leads to accumulation of Slug (Snail2). The accumulated Slug targets LSD1 to the promoter region of *BRCA1* gene, and subsequent suppression of BRCA1 expression via histone modification [6]. However it is not known whether overexpression of LSD1 directly affects BRCA1 expression.

**Fig 4:**
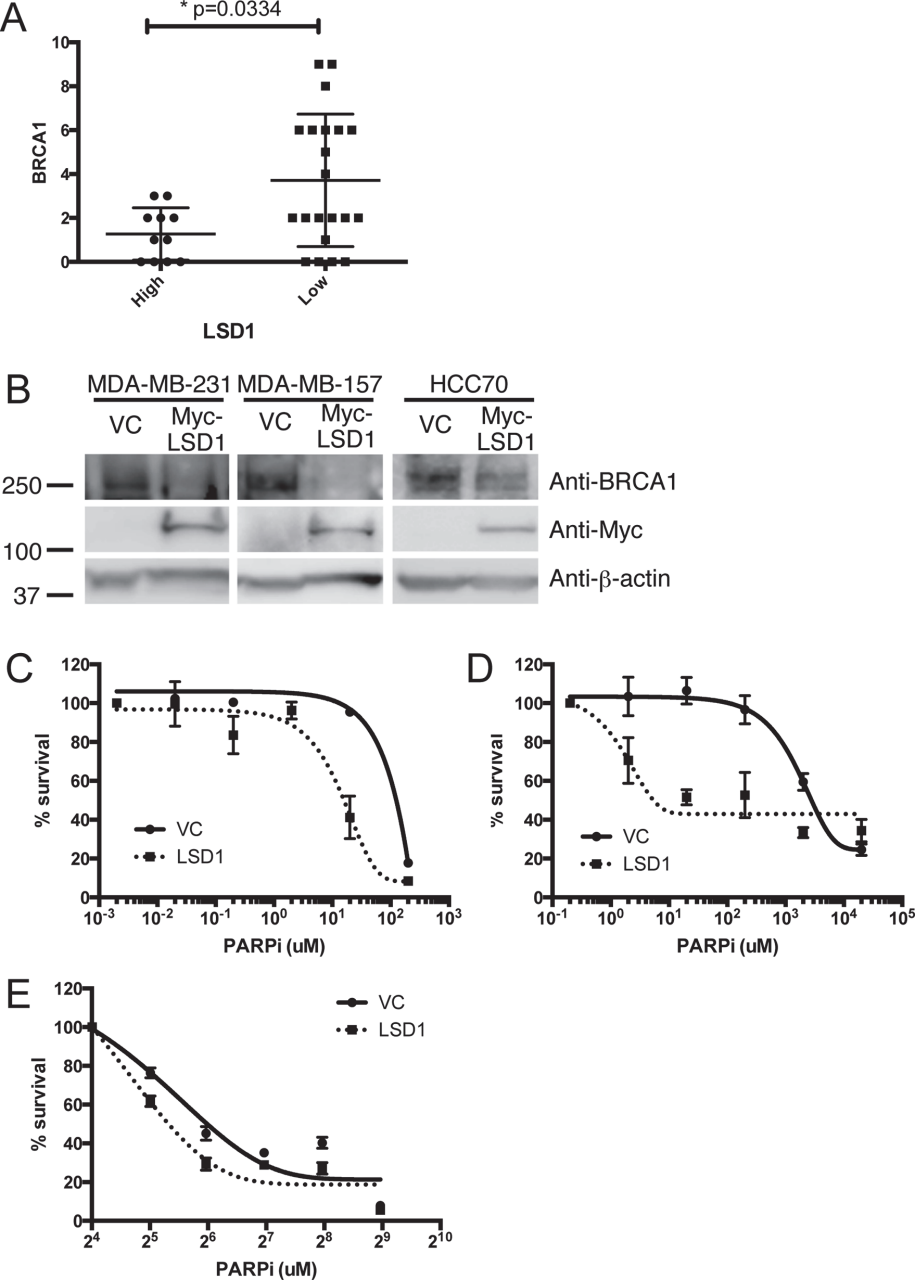
Overexpression of LSD1 reduces BRCA1 expression in triple negative or in basal-like breast cancer and increases sensitivity to PARP inhibitor in basal-like breast cancer. Histogram shows protein expression of BRCA1 in triple negative breast cancer with high or low level of LSD1 protein (A). Y-axis indicates IHC score of BRCA1. Basal-like breast cancer cell lines (MDA-MB-231, MDA-MB-157 and HCC70) was transfected as indicated. Cell lysates were subjected for Western Blots with indicated antibodies (B). Cell viability of basal-like breast cancer cell lines are assessed by colony formation assay. Percent survival is shown in different doses of PARP inhibitor. Error bars show standard deviation of three independent experiments (C-E).

We therefore analyzed the relationship between *BRCA1* and *LSD1* protein level in our set of triple negative breast cancer samples. Eleven cases exhibit high level of LSD1 (IHC score > 5) and have significantly decreased expression level of BRCA1 in comparison to TNBC samples with low/normal LSD1 levels (p = 0.0334) (Table 4 and Fig. 4A). We then tested the effect of ectopic expression of *LSD1* in a set of 3 basal-like breast cancer cell lines. Overexpression of *LSD1* suppresses endogenous expression of BRCA1 in all three cell lines we tested (Fig. 4B).

PARP inhibitors are a novel class of potent anti-cancer agents with specificity for cancers with mutant *BRCA* genes, through a synthetic lethal relationship [26]. Further studies have revealed that sensitivity to PARP inhibitor is predicted not only by mutations in the *BRCA* genes, but also by low expression levels of the BRCA1 protein [15]. As shown in Fig. 4A-B and Table 4, overexpression of LSD1 suppresses expression of BRCA1. Therefore we hypothesized that basal-like breast cancer cells with overexpression of *LSD1* may be sensitive to PARP inhibitor via suppression of BRCA1. Accordingly, ectopic expression of LSD1 leads to an increased sensitivity of basal-like breast cancer cells to PARP inhibition (Fig. 4C-E). Thus, we propose that PARP inhibition may be a potential therapeutic strategy for basal-like breast cancer, especially in the poor prognosis subgroup as defined by overexpression of *LSD1*.

## Discussion and Conclusions

We have investigated the impact of *LSD1* expression on clinical outcome in a large data set of breast cancer. We found that amplification of *LSD1* is observed in basal-like breast cancer, and is a prognostic factor of poor outcome in this subtype. We have also found that protein product of *LSD1* is a biomarker of poor outcome in triple negative breast cancer. There have been several studies attempting to develop methods to diagnose poor prognostic subgroups of triple negative and basal-like breast cancer, because of the poor clinical outcome associated with this subtype despite aggressive chemotherapy in the adjuvant setting. The results shown here suggest that LSD1 levels could potentially serve as such a marker. Since the work presented is based on retrospective and *in-vitro* analyses, further prospective validation in clinical samples is needed.

We have found that LSD1 overexpression suppresses levels of BRCA1 in our clinical samples of triple negative breast cancer, and that this effect is reproduced in *in-vitro* models. Wnt signaling has been reported to modulate the link between LSD1 and BRCA1 [6]. Wnt signaling is upregulated in several types of cancer, and this may suggest that suppression of BRCA1 by LSD1 could play a role in other type of cancers as well [27], where LSD1 overexpression has been reported to be a prognostic factor of poor clinical outcome [7] [8] [9] [10]. Further studies *in-vitro* and *in-vivo* will be required to confirm this.

Since it has been reported that suppression of BRCA1 sensitizes cells to PARP inhibitors, we speculated that LSD1 overexpressing cancer cells would be sensitive to PARP inhibition [15]. As expected, indeed *LSD1* overexpressing cancer cells were sensitive to this drug *in vitro*. Subsequent validation of these findings in a larger clinical cohorts and cell line collections will be required for development of this finding into a clinically applicable biomarker. Nonetheless, our findings are the first to demonstrate that LSD1 is a prognostic factor in basal-like breast cancer and PARP inhibition is a potentially an effective therapeutic strategy for this difficult to treat subtype of cancer.

## Supporting Information

S1 Fig High-expression of LSD1 is linked to poor outcome in basal-like breast cancer. Effect of high LSD1 (as defined as values >median) on recurrence free survival (RFS) in all or in specific subtypes of breast cancer are shown (overall breast cancer, n = 3180 (A), basal-like, n = 540 (B), Lumina A, n = 1540 (C), Luminal B, n = 907 (D) and HER2, n = 193 (E)) using pooled data from kmplot.org. High expression of LSD1 transcripts shows significant shorter RFS in all or basal-like breast cancer (p = 0.0015, p = 0.0025, respectively). KMplot divides the data into two groups based on LSD1 expression as compared to the median. P-value was calculated using Gehan-Breslow-Wilcoxon test.
(TIF)
