# Supplementary figures and images for: LSD1 Overexpression Is Associated with Poor Prognosis in Basal-Like Breast Cancer, and Sensitivity to PARP Inhibition
Source: PLoS One. 2015 Feb 13;10(2):e0118002. doi: 10.1371/journal.pone.0118002 (PMC4332491; doi:10.1371/journal.pone.0118002)

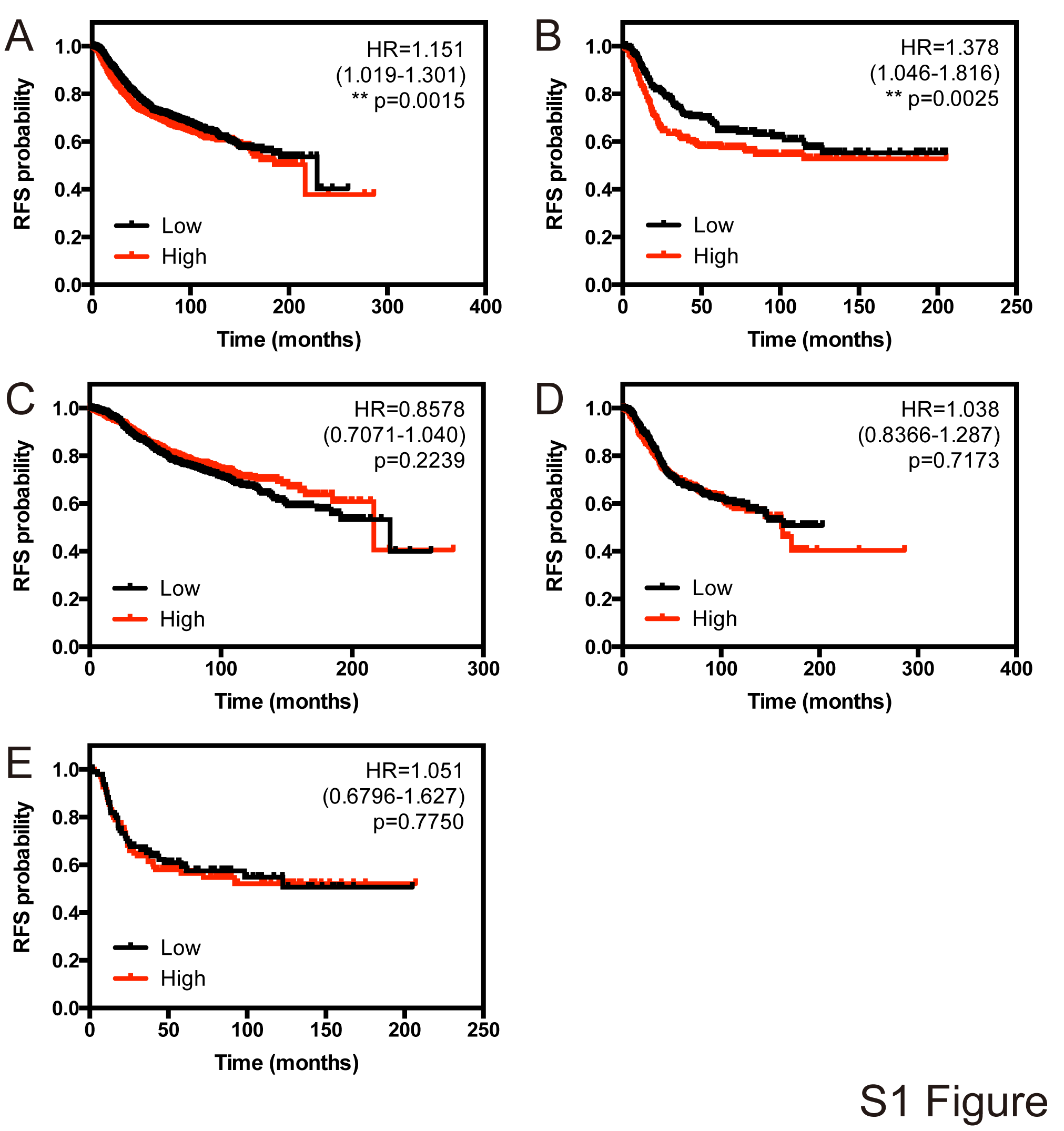

Supplement: S1 Fig — Effect of high LSD1 (as defined as values >median) on recurrence free survival (RFS) in all or in specific subtypes of breast cancer are shown (overall breast cancer, n = 3180 (A), basal-like, n = 540 (B), Lumina A, n = 1540 (C), Luminal B, n = 907 (D) and HER2, n = 193 (E)) using pooled data from kmplot.org. High expression of LSD1 transcripts shows significant shorter RFS in all or basal-like breast cancer (p = 0.0015, p = 0.0025, respectively). KMplot divides the data into two groups based on LSD1 expression as compared to the median. P-value was calculated using Gehan-Breslow-Wilcoxon test. (TIF) [file pone.0118002.s001.tif]
